# Life’s essential 8, genetic susceptibility, and risk of inflammatory bowel diseases: a population-based cohort study

**DOI:** 10.1186/s12966-024-01617-3

**Published:** 2024-07-02

**Authors:** Honghao Yang, Qing Chang, Chao Ji, Gang Zheng, Zheng Ma, Liangkai Chen, Yang Xia, Yuhong Zhao

**Affiliations:** 1 Department of Clinical Epidemiology, Shengjing Hospital of China Medical University, No. 36, San Hao Street, Shenyang, Liaoning 110004 China; 2 Liaoning Key Laboratory of Precision Medical Research on Major Chronic Disease, Shenyang, Liaoning Province China; 3 Clinical Research Center, Shengjing Hospital of China Medical University, Shenyang, China; 4 Department of Nutrition and Food Hygiene, Hubei Key Laboratory of Food Nutrition and Safety, School of Public Health, Tongji Medical College, Huazhong University of Science and Technology, Wuhan, China

**Keywords:** Life’s essential 8, IBD, UC, CD, CVH

## Abstract

**Background:**

Evidence has shown that the individual metrics in Life’s Essential 8 (LE8), an updated cardiovascular health (CVH) concept proposed by the American Heart Association, play a role in the development of inflammatory bowel disease (IBD). However, epidemiological evidence on the overall LE8 on IBD risk remains limited. We aimed to assess the longitudinal associations of LE8-defined CVH and the risks of IBD and its subtypes, ulcerative colitis (UC) and Crohn’s disease (CD). We also tested whether genetic susceptibility could modify these associations.

**Methods:**

A total of 260,836 participants from the UK Biobank were included. LE8 scores were determined by 8 metrics (physical activity, diet, nicotine exposure, sleep, body mass index, blood pressure, blood glucose, and blood lipids), and were divided into three levels: low CVH (0–49), moderate CVH (50–79), and high CVH (80–100). Cox proportional hazards models were used to calculate the hazard ratios (HRs) and confidence intervals (CIs) of the risk of IBD in relation to CVH status.

**Results:**

Over a median follow-up 12.3 years, we documented 1,500 IBD cases (including 1,070 UC and 502 CD). Compared to participants with low CVH, the HRs (95% CIs) of those with high CVH for IBD, UC, and CD were 0.67 (0.52, 0.83), 0.70 (0.52, 0.93), and 0.55 (0.38, 0.80), respectively. These associations were not modified by genetic susceptibility (all *P* for interactions > 0.05). The lowest HR (UC: 0.30, 95% CI: 0.20–0.45; CD: 0.33, 95% CI: 0.20–0.57) was observed in participants with both high CVH and low genetic risk.

**Conclusions:**

Better CVH, defined by LE8, was associated with significantly lower risks of IBD, UC, and CD, irrespective of genetic predisposition. Our results underscore the importance of adherence to LE8 guidelines for maintaining CVH as a potential strategy in the prevention of IBD.

**Supplementary Information:**

The online version contains supplementary material available at 10.1186/s12966-024-01617-3.

## Background

Inflammatory bowel diseases (IBD), encompassing two subtypes (ulcerative colitis [UC] and Crohn’s disease [CD]), are chronic conditions affecting the digestive system [1]. In 2019, there were an estimated 4.9 million IBD patients globally [1]. These conditions are principally characterized by intestinal ulcers and manifest with debilitating symptoms including bloody diarrhea, abdominal pain, and fever, severely impairing patients’ quality of life [2, 3]. Furthermore, IBD also imposes high financial costs to patients and society [4]. Given its widespread prevalence and the challenges associated with its management, IBD has been a substantial global health concern [5]. Therefore, investigating modified risk factors is pivotal for IBD prevention.

A recently updated cardiovascular health (CVH) concept proposed by the American Heart Association (AHA), known as Life’s Essential 8 (LE8) [6], has been identified as a potent predictor of risk of cardiovascular disease (CVD) and other chronic conditions, such as nonalcoholic fatty liver disease and asthma [7–10]. Based on the Life’s Simple 7, LE8 incorporates ideal health behaviors (less nicotine exposure, body mass index [BMI] < 25 kg/m^2^, ideal physical activity [PA], and healthy diets based on guideline recommendations) and health factors (normal levels of serum lipids, blood pressure [BP], and blood glucose), with the addition of sleep health [6].

Accumulated evidence has shown that IBD patients are at a higher risk of CVD, potentially due to shared pathophysiological mechanisms [11]. Thus, it is possible that promoting CVH may be beneficial for the prevention of IBD. In addition, previous studies have demonstrated that the individual CVH metrics in LE8 play an important role of development of IBD [12, 13]. Notably, these LE8 factors are interrelated, and improving one might lead to changes to others. For example, optimal PA and a balanced diet might lead to a healthier BMI and normalize serum lipids levels [14–16]. Therefore, it is essential to consider the combination of these multiple LE8 factors, which may affect IBD in a concerted manner. However, research focusing on the association of LE8 with the risk of IBD is limited.

Furthermore, a hereditary component in the development of IBD is well-recognized and a polygenic predisposition to IBD has been identified [17]. Examining the interplay between genetic susceptibility to IBD and LE8 could help identify those more predisposed to IBD, for whom following LE8 guidelines might offer greater benefits in preventing the onset of this condition.

In the present study, we aimed to investigate the longitudinal associations of the levels of CVH, defined by LE8 scores, with the risks of IBD and its two subtypes UC and CD among participants from the UK biobank. We also examined the interactions between genetic predisposition to UC or CD and LE8 scores, as well as the joint association of LE8 scores and genetic factors with the risk of UC or CD.

## Methods

### Study design and population

This study utilized data from the UK Biobank, which is a population-based cohort comprising over 500,000 participants aged between 37 and 73 years [18]. Participants attended at one of 22 centers across England, Scotland, and Wales between 2006 and 2010. At assessment center, participants were needed to provide biological samples, complete touch-screen questionnaires, and undergo physical examination. This study was approval by National Information Governance Board for Health and Social Care and the National Health Service North West Multicenter Research Ethics Committee (REC reference: 21/NW/0157).

In this study, we included participant with complete data on LE8 metric at baseline (*n* = 281,190). After excluding participant who lack of genetic information or with a mismatch between genetic sex and self-reported gender (*n* = 2,549), were non-European ancestry (*n* = 12,574), or had missing data on any covariates (*n* = 1,732), with a history of IBD (*n* = 2,829), or lost to follow-up (*n* = 670), 260,836 individuals remained in our final study sample (flowchart of participant selection, Supplementary Figure 1).

### CVH assessment with LE8

According to the AHA’s definition, LE8 includes health behaviors (diet, nicotine exposure, PA, BMI, and sleep) and health factors (BP, blood glucose, and non-high-density-lipoprotein [HDL]-cholesterol) [6]. Dietary information was assessed by a validated food frequency questionnaire (FFQ) [19]. Based on the responses to this FFQ, dietary quality was assessed through a healthy diet score (HDS) [20], which combined several foods in terms of quantity and frequency of consumption per week. Supplementary Table 1 details the selected food groups, their coding scheme, and the scoring system used to construct HDS [20]. In brief, a total of 13 food groups were selected. Participants received 1 point for each food group consumed at an ideal level, or 0 points if not. HDS was calculated as the sum of points from the 13 food groups, with higher scores indicating better dietary quality. Data on nicotine exposure, sleep duration, frequency and duration of moderate and vigorous PA, and medications use for managing BP, blood lipids, and blood glucose were collected by touch screen questionnaires at baseline. Height and weight were assessed by trained staff, and BMI was calculated as weight (kg)/height (m^2^). BP was measured twice using an Omron device, with the average systolic and diastolic values used for analysis. Total cholesterol was quantified through the cholesterol oxidase-peroxidase method, while HDL-cholesterol was measured using enzyme immunoinhibition on a Beckman Coulter AU5800 [21]. Glycated hemoglobin levels were determined by high-performance liquid chromatography on a Bio-Rad VARIANT II Turbo [21].

Each component was assigned a score ranging from 0 to 100 points, with detailed definitions and scoring criteria provided in the Supplementary Tables 1 and 2. The LE8 scores were calculated by averaging the 8 metrics. Following the AHA’s advisory [6], we categorized CVH into three levels based on the LE8 scores: high CVH (80–100), moderate CVH (50–79), and low CVH (0–49).

### Assessment of outcomes

The disease outcomes were defined as primary or secondary events using inpatient hospital and death registry data linked to the UK Biobank. IBD was defined as International Classification of Diseases (ICD) 9th edition (ICD-9) codes 555 (CD) and 556 (UC), as well as edition 10 (ICD-10) codes K50 (CD) and K51 (UC). Person-years of follow-up were calculated from the recruitment date to the earliest occurrence of an IBD diagnosis, death, or the end of follow-up (September 30, 2021 for centers in England; February 28, 2018, for centers in Wales; and July 31, 2021, for centers in Scotland).

### Calculation of polygenic risk scores

The polygenic risk scores (PRS) were calculated by the UK Biobank team [22]. The Genome-Wide Association Studies summary statistics for these PRSs are available at https://zenodo.org/record/6631952. These PRSs underwent validation using participants from the UK Biobank and underwent four rounds of comprehensive validation through the Electronic Medical Records and Genomics Network. The UK Biobank filed IDs for CD-PRS and UC-PRS were 26,229 and 26,287, respectively. In our analysis of PRS, we classified PRS as low (< median) and high (≥ median) risks.

### Assessment of covariates

Sociodemographic information (including age, sex, and education) and alcohol drinking status were obtained from touchscreen questionnaires. Education levels were classified into three categories: high (College or University degree), medium (A levels/AS levels or equivalent, NVQ, HND, HNC, or equivalent, and other professional qualifications), and low (CSEs or equivalent, O levels/GCSEs or equivalent, or none of the above). Participants reported their alcohol consumption status as either never, previous, or current drinkers. The Townsend Deprivation Index (TDI), derived from the participant’s postcode of residence, was used as an area-based proxy measure for socioeconomic status [23]. The prevalent depression at baseline was collected through the self-reported medical history (field ID: 20,002, codes: 1286, 1291, and 1531) or hospitalization records (ICD-10 codes: F32 and F33).

### Statistical analysis

Baseline characteristics were summarized across CVH levels as percentage for categorical variables and mean and standard deviation (SD) for continuous variables. The differences of these variables across CVH levels were examined using the analysis of covariance for continuous variables and the Chi-square test for categorical variables. Cox proportional hazards regression models were fit to estimate the hazard ratios (HRs) and 95% confidence intervals (95% CIs) for the associations of LE8 scores (as low, moderate, and high levels of CVH or per 10-point increase) with the risks of IBD, UC, and CD, separately. The Schoenfeld residuals method and the Kaplan–Meier method were used to test the proportional hazards assumption and no violations of this assumption were observed. Three models were built. Model 1 was a crude model. Model 2 was adjusted for age (continuous) and sex (male or female). Model 3 was further adjusted for TDI (categorical, quartiles), drinking status (current, previous, or never), education level (low, medium, or high), depression (yes or no), UC-PRS (low or high, only in UC), CD-PRS (low or high, only in CD), genotyping batch (only in UC and CD), and the first 10 principal components of genetics (only in UC and CD). Additionally, restricted cubic spline regression was used to explore the dose-response associations between the LE8 scores and the risks of IBD, UC and CD. Four knots were automatically selected based on the minimum Akaike Information Criterion, with the 10th percentile of the LE8 scores set as the reference point and all relevant covariates adjusted. In addition, we assessed the associations between individual components of LE8 (each SD increase) and the risk of IBD with further adjusting for the overall LE8 score (each SD increase) calculated from the remaining 7 components.

We further investigated the potential genetic risk-modifying effects on the associations of LE8 scores with the risks of UC and CD. The interactions between LE8 scores and PRSs were calculated by adding interaction terms of LE8 scores and UC-PRS or CD-PRS in the final Cox model (model 3). Furthermore, we also assessed the combined effect of LE8 scores and PRSs on the risks of UC and CD. Specifically, we categorized participants into six groups based on the combination of LE8 scores and PRS categories, with the low level of LE8 scores (low CVH) and high PRSs as the reference category.

In addition, we performed strata analyses to examine whether sex and age (< 55 or ≥ 55 years) modified the association of LE8 scores with IBD risk. We assessed the potential modification effects by introducing interaction terms of LE8 scores and the stratifying variables. Furthermore, a series of sensitivity analyses were performed to assess the robustness of our findings. First, we excluded participants with a history of cancer at baseline due to potential impacts of this condition on their CVH factors. Second, we excluded participants diagnosed with IBD within the first 2 years of follow-up to mitigate the potential impact of reverse causality. Third, we further utilized the Fine–Gray sub-distribution hazard model to account for the potential impact of mortality as a competing event.

Data cleaning and analyses were performed using SAS version 9.4 (SAS Institute Inc., Cary, NC, USA). A two-sided p-value of < 0.05 was considered statistically significant.

## Results

### Baseline characteristics

Table 1 presents the baseline characteristics of participants according to low, moderate, and high levels of CVH defined by LE8 scores. Among 260,836 participants, 17.7% of those achieved a high level of CVH, whereas 5.71% had a low level. Compared to those with low CVH, percipients with high CVH were more likely to be females, younger, and less economically deprived, had a higher education level, tended to be current drinkers, and had a lower proportion of depression at baseline.

**Table 1:** Baseline characteristics of participants according to Life’s Essential 8 scores ^a^

Characteristics	Life’s Essential 8			*P* value ^b^
	Low (0–49)	Moderate (50–79)	High (80–100)	
Participants, *n*	14,899 (5.71)	199,905 (76.6)	46,032 (17.7)	-
Age (years)	56.6 (7.54) ^c^	57.0 (7.89)	53.5 (8.26)	< 0.0001
Sex				< 0.0001
Females	6,020 (40.4)	97,981 (49.0)	31,444 (68.3)	
Males	8,879 (59.6)	101,924 (51.0)	14,588 (31.7)	
TDI	-0.44 (3.37)	-1.60 (2.91)	-1.87 (2.73)	< 0.0001
Education level				< 0.0001
Low	7,505 (50.4)	82,281 (41.2)	15,647 (34.0)	
Medium	6,263 (42.0)	95,255 (47.6)	23,370 (50.8)	
High	1,131 (7.59)	22,369 (11.2)	7,015 (15.2)	
Drinking status				< 0.0001
Current	13,510 (90.7)	187,562 (93.8)	43,155 (93.8)	
Previous	892 (5.99)	6,306 (3.15)	1,281 (2.78)	
Never	497 (3.34)	6,037 (7.02)	1,596 (3.47)	
Depression				< 0.0001
Yes	1,783 (12.0)	12,069 (6.04)	2,172 (4.72)	
No	13,116 (88.0)	187,836 (93.9)	43,860 (95.3)	
**Life’s Essential 8 components**				
BMI (kg/m^2^)	32.6 (5.73)	27.7 (4.39)	23.7 (2.67)	< 0.0001
Healthy diet score	3.19 (1.58)	4.39 (1.66)	5.49 (1.49)	< 0.0001
Moderate PA (min/wk)	77.3 (278.2)	277.8 (465.0)	326.8 (452.8)	< 0.0001
Vigorous PA (min/wk)	21.0 (129.6)	88.6 (199.2)	125.9 (189.0)	< 0.0001
Sleep duration (hours/day)	6.89 (1.69)	7.16 (1.06)	7.32 (0.77)	< 0.0001
SBP (mmHg)	147.4 (17.5)	140.3 (17.8)	123.4 (13.9)	< 0.0001
DBP (mmHg)	88.5 (10.1)	83.5 (9.67)	74.6 (8.05)	< 0.0001
Non-HDL cholesterol (mg/dL)	184.5 (46.5)	169.0 (40.7)	139.0 (30.9)	< 0.0001
Glycated haemoglobin (%)	3.76 (1.10)	3.28 (0.55)	3.10 (0.34)	< 0.0001

^a^ Abbreviations: BMI, body mass index; DBP, diastolic blood pressure; HDL, high-density-lipoprotein; PA, physical activity; SBP, systolic blood pressure; TDI, Townsend Deprivation Index

^b^ Analysis of covariance or Chi-square test

^c^ Continuous variables were presented as mean (standard deviation) and categorical variables were shown as percentage

### LE8 and IBD, UC, and CD

**Fig. 1:**
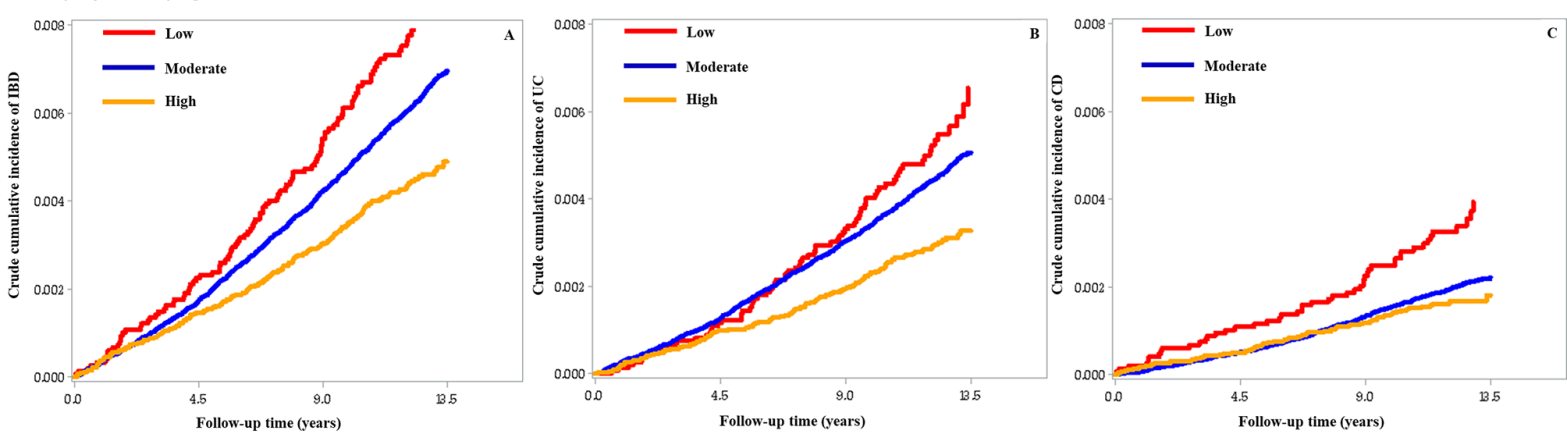
Crude cumulative incidence of IBD **(A)**, UC **(B)**, and CD **(C)** according to categories of Life’s Essential 8 scores. Participants were categorized into three cardiovascular health groups according to Life’s Essential 8 scores: low (0–49), moderate (50–79), and high (80–100). Abbreviations: CD, Crohn’s disease; IBD, inflammatory bowel disease; UC, ulcerative colitis

During a median follow-up of 12.3 years, 1,500 incident IBD cases were documented, including 1,070 UC and 502 CD. The cumulative incidence showed graded relationships according to the levels of CVH for IBD, UC and CD during follow-up (*P* < 0.001 for all log-rank tests, Fig. 1). Table 2 shows the associations between the LE8 scores and the incidence of IBD, UC, and CD. Compared to the low CVH level, moderate (HR: 0.82; 95% CI: 0.67–0.99) and high (HR: 0.67; 95% CI: 0.52–0.83) levels of CVH were significantly associated with lower risk of developing IBD in the fully adjusted model. Similar results were observed in the associations of LE8 scores with UC and CD risks. The fully-adjusted HRs (95% CIs) for UC across low, moderate, and high CVH levels were 1.00 (reference), 0.92 (0.73, 1.17), and 0.70 (0.52, 0.93), respectively; for CD, the corresponding HRs (95% CIs) were 1.00 (reference), 0.63 (0.46, 0.85), and 0.55 (0.38, 0.80), respectively. Additionally, each 10-point increase in LE8 scores was significantly associated with reduced risks of developing IBD (HR: 0.90; 95% CI: 0.86–0.94), UC (HR: 0.90; 95% CI: 0.85–0.95), and CD (HR: 0.88; 95% CI: 0.82–0.95). As presented in Supplementary Table 3, the fully adjusted HRs (95% CIs) of incident IBD, UC, and CD for each SD increase in the LE8 components were: 0.94 (0.89, 0.99), 0.95 (0.90, 1.01), and 0.92 (0.84, 1.00) for dietary quality score; 0.93 (0.89, 0.98), 0.96 (0.90, 1.01), and 0.88 (0.81, 0.95) for PA score; 0.92 (0.88, 0.96), 0.94 (0.89, 0.99), and 0.87 (0.81, 0.94) for sleep health score; 0.88 (0.84, 0.92), 0.89 (0.85, 0.95), and 0.83 (0.77, 0.90) for nicotine exposure score; 0.93 (0.89, 0.98), 0.92 (0.87, 0.98), and 0.95 (0.87, 1.04) for BMI score; 1.16 (1.10, 1.22), 1.11 (1.04, 1.17), and 1.26 (1.16, 1.38) for blood lipids score; 0.95 (0.91, 0.99), 0.95 (0.90, 0.99), and 0.99 (0.91, 1.07) for blood glucose score; and 1.02 (0.96, 1.08), 0.99 (0.93, 1.06), and 1.06 (0.97, 1.17) for BP score, respectively.

**Table 2:** Associations of life’s essential 8 scores with the risk of IBD, UC, and CD ^a^

	Life’s Essential 8			Per 10-point increase
	Low (0–49)	Moderate (50–79)	High (80–100)	
**IBD**				
Cases/participants, n	114/14,899	1,187/199,905	199/46,032	1,500/260,836
Person-years	172,127	2,352,673	544,541	3,069,341
Model 1 ^c^	1.00 (reference)	0.76 (0.63, 0.92) ^b^	0.55 (0.44, 0.69)	0.86 (0.83, 0.90)
Model 2 ^d^	1.00 (reference)	0.77 (0.64, 0.93)	0.61 (0.48, 0.77)	0.88 (0.84, 0.92)
Model 3 ^e^	1.00 (reference)	0.82 (0.67, 0.99)	0.67 (0.52, 0.83)	0.90 (0.86, 0.94)
**UC**				
Cases/participants, n	75/14,899	862/199,905	133/46,032	1,070/260,836
Person-years	172,357	2,354,472	544,969	3,071,798
Model 1	1.00 (reference)	0.84 (0.66, 1.06)	0.56 (0.42, 0.74)	0.86 (0.81, 0.90)
Model 2	1.00 (reference)	0.86 (0.68, 1.09)	0.64 (0.48, 0.84)	0.88 (0.84, 0.93)
Model 3	1.00 (reference)	0.92 (0.73, 1.17)	0.70 (0.52, 0.93)	0.90 (0.85, 0.95)
**CD**				
Cases/participants, n	48/14,899	380/199,905	74/46,032	502/260,836
Person-years	172,449	2,357,183	545,251	3,074,883
Model 1	1.00 (reference)	0.58 (0.43, 0.78)	0.49 (0.34, 0.70)	0.86 (0.80, 0.92)
Model 2	1.00 (reference)	0.58 (0.43, 0.78)	0.49 (0.34, 0.71)	0.86 (0.80, 0.93)
Model 3	1.00 (reference)	0.63 (0.46, 0.85)	0.55 (0.38, 0.80)	0.88 (0.82, 0.95)

^a^ Abbreviations: CD, Crohn’s disease; IBD, inflammatory bowel disease; PRS, polygenic risk scores; UC, ulcerative colitis

^b^ Hazard ratio (95% confidence interval) (all such values)

^c^ Model 1 was a crude model

^d^ Model 2 was adjusted for age (continuous) and sex (male or female)

^e^ Model 3 was further adjusted for Townsend Deprivation Index (categorical, quartiles), drinking status (current, previous, or never), education levels (low, medium, or high), depression (yes or no), UC-PRS (< median or ≥ median, only in UC), CD-PRS (< median or ≥ median, only in CD), genotyping batch (only in UC and CD), and the first 10 principal components of genetics (only in UC and CD)

**Fig. 2:**
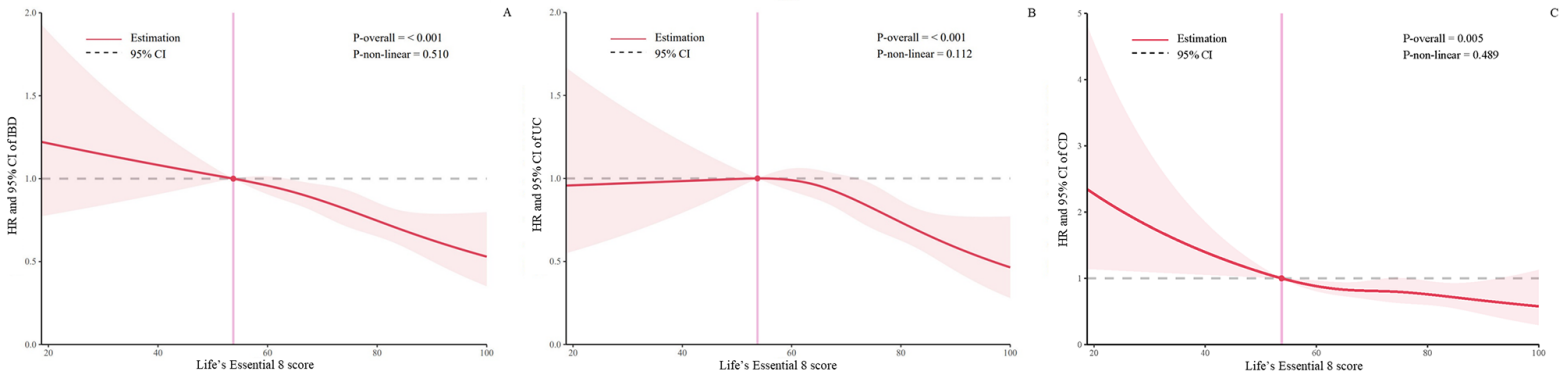
The dose–response associations of the Life’s Essential 8 scores with the risks of IBD **(A)**, UC **(B)**, and CD **(C)**. Multivariable Cox proportional hazards regression was adjusted for age (continuous), sex (male or female), Townsend Deprivation Index (categorical, quartiles), drinking status (current, previous, or never), education levels (low, medium, or high), depression (yes or no), UC-PRS (< median or ≥ median, only in UC), CD-PRS (< median or ≥ median, only in CD), genotyping batch (only in UC and CD), and the first 10 principal components of genetics (only in UC and CD). Four knots were automatically selected based on the minimum Akaike Information Criterion, with the 10th percentile of the Life’s Essential 8 scores set as the reference point. Abbreviations: CD, Crohn’s disease; CI, confidence interval; HR, hazard ratio; IBD, inflammatory bowel disease; PRS, polygenic risk scores; UC, ulcerative colitis

Figure 2 displays the dose–response associations of the LE8 scores with the risks of IBD, UC, and CD. All of these associations appeared to be linear (all *P* for non-linear > 0.05).

### Effect modification by genetic susceptibility

**Fig. 3:**
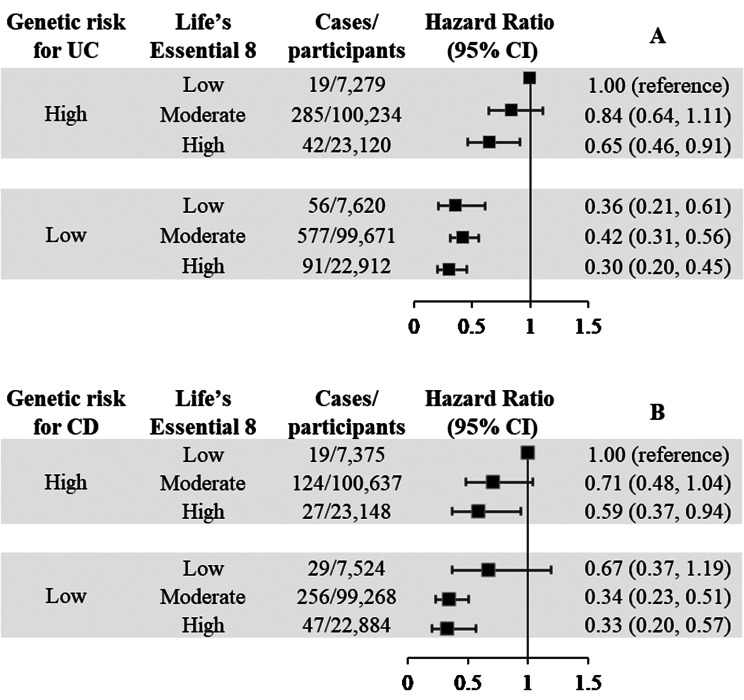
The joint associations of Life’s Essential 8 scores and PRSs with the risk of UC **(A)** and CD **(B)**. Multivariable Cox proportional hazards regression was adjusted for age (continuous), sex (male or female), Townsend Deprivation Index (categorical, quartiles), drinking status (current, previous, or never), education levels (low, medium, or high), depression (yes or no), genotyping batch (only in UC and CD), and the first 10 principal components of genetics (only in UC and CD). For CVH, participants were categorized into three CVH groups based on Life’s Essential 8 scores: low (0–49), moderate (50–79), and high (80–100). For genetic risk, participants were stratified into two groups based on the medians of UC-PRS or CD-PRS (< median: low genetic risk; ≥ median: high genetic risk). Abbreviations: CD, Crohn’s disease; CI, confidence interval; CVH, cardiovascular health; PRS, polygenic risk scores; UC, ulcerative colitis

A higher UC-PRS or CD-PRS was associated with a higher risk of UC or CD (Supplementary Table 4). Participants with high genetic risk had 107% (HR: 2.07; 95% CI: 1.82–2.36) and 97% (HR: 1.97; 95% CI: 1.64–2.37) higher risk of UC and CD, respectively (Supplementary Table 4). There was no evidence of multiplicative interaction between LE8 scores and UC-PRS (*P* for interaction = 0.65) or CD-PRS (*P* for interaction = 0.83) (Table 3). Figure 3 shows the joint associations of PRSs and LE8 scores with incident UC and CD. Compared to participants in the high PRS and low CVH category, the lowest HR (HR for UC: 0.30; 95% CI: 0.20–0.45; HR for CD: 0.33; 95% CI: 0.20–0.57) was observed for those with low PRS combined with the high CVH.

**Table 3:** Associations of life’s essential 8 scores with the risk of UC and CD according to genetic risk ^a^

	Life’s Essential 8			Per 10-point increase	*P* for interaction ^b^
	Low (0–49)	Moderate (50–79)	High (80–100)		
**UC**					0.65
Low genetic risk					
Cases/participants, *n*	19/7,279	285/100,234	42/23,120	346/130,633	
Person-years	84,086	1,179,543	273,485	1,537,114	
Model 1 ^d^	1.00 (reference)	1.07 (0.67, 1.70) ^c^	0.68 (0.40, 1.17)	0.84 (0.77, 0.92)	
Model 2 ^e^	1.00 (reference)	1.10 (0.69, 1.76)	0.80 (0.46, 1.38)	0.88 (0.80, 0.96)	
Model 3 ^f^	1.00 (reference)	1.19 (0.74, 1.89)	0.88 (0.51, 1.53)	0.89 (0.81, 0.98)	
High genetic risk					
Cases/participants, *n*	56/7,620	577/99,671	91/22,912	724/130,203	
Person-years	88,270	1,174,929	271,483	1,534,682	
Model 1	1.00 (reference)	0.77 (0.59, 1.02)	0.53 (0.38, 0.74)	0.86 (0.81, 0.92)	
Model 2	1.00 (reference)	0.78 (0.60, 1.03)	0.59 (0.42, 0.82)	0.89 (0.83, 0.94)	
Model 3	1.00 (reference)	0.83 (0.63, 1.09)	0.62 (0.44, 0.88)	0.90 (0.84, 0.96)	
**CD**					0.83
Low genetic risk					
Cases/participants, *n*	19/7,375	124/100,637	27/23,148	170/131,160	
Person-years	85,422	1,186,348	274,128	1,545,898	
Model 1	1.00 (reference)	0.47 (0.29, 0.76)	0.44 (0.25, 0.79)	0.83 (0.73, 0.94)	
Model 2	1.00 (reference)	0.47 (0.29, 0.77)	0.45 (0.25, 0.82)	0.83 (0.73, 0.94)	
Model 3	1.00 (reference)	0.51 (0.31, 0.83)	0.51 (0.28, 0.93)	0.85 (0.75, 0.97)	
High genetic risk					
Cases/participants, *n*	29/7,524	256/99,268	47/22,884	332/129,676	
Person-years	87,027	1,170,835	271,123	1,528,985	
Model 1	1.00 (reference)	0.66 (0.45, 0.96)	0.52 (0.33, 0.82)	0.87 (0.80, 0.96)	
Model 2	1.00 (reference)	0.65 (0.44, 0.95)	0.52 (0.33, 0.84)	0.87 (0.80, 0.96)	
Model 3	1.00 (reference)	0.70 (0.48, 1.04)	0.58 (0.36, 0.94)	0.90 (0.81, 0.98)	

^a^ Abbreviations: CD, Crohn’s disease; PRS, polygenic risk scores; UC, ulcerative colitis

^b^*P* for interaction was assessed by adding the multiplicative interaction terms of LE8 scores with PRS in the models

^c^ Hazard ratio (95% confidence interval) (all such values)

^d^ Model 1 was a crude model

^e^ Model 2 was adjusted for age (continuous) and sex (male or female)

^f^ Model 3 was further adjusted for Townsend Deprivation Index (categorical, quartiles), drinking status (current, previous, or never), education levels (low, medium, or high), depression (yes or no), genotyping batch, the first 10 principal components of genetics, UC-PRS (continuous, only in UC), and CD-PRS (continuous, only in CD)

### Subgroup and sensitivity analyses

In the stratified analyses, the negative associations of LE8 scores with the risks of IBD, UC, and CD were consistent across strata by age and sex (all *P* for interactions > 0.05) (Supplementary Table 5). The same association pattern was observed in a series of sensitivity analyses with exclusions of participants with baseline cancer (Supplementary Table 6) or developing IBD within the first 2 years of follow-up (Supplementary Table 7), or the use of Fine–Gray competing risk regression (Supplementary Table 8).

## Discussion

Based on this large-scale cohort study, we found that better CVH, defined as higher LE8 scores, was associated with a decreased risk of IBD and its subtypes, UC and CD. These associations remained consistent across different genetic risk categories for UC and CD, with no significant interactions. Furthermore, the lowest risk of UC or CD was observed in participants with a combination of low genetic risk and high CVH, compared to those with high genetic risk and low CVH.

CVD is a well-established comorbidity of IBD [24]. Previous studies have suggested the protective role of maintaining optimal CVH metrics for the prevention of IBD. For examples, adhering to a healthy diet [25, 26], smoking cessation [27], participating in regular and enough PA [12], and keeping a normal BMI [28] have been associated with a reduced risk of developing IBD. Our findings partially aligned with these observations. Specifically, we found that reducing nicotine exposure and maintaining adequate sleep duration were significantly associated with lower risks of developing IBD, UC, and CD. However, our analysis revealed that not all individual components of the LE8 (e.g., BP) demonstrated a significantly protective association with the risk of IBD. In particular, higher blood lipid scores were paradoxically linked to increased risks of IBD, UC, and CD. These findings can be partially explained as follows. On the one hand, individuals diagnosed with metabolic disorders often adopt healthier lifestyles post-diagnosis [29, 30], which may confound the observed associations. On the other hand, medications commonly used to manage these conditions, such as statins, have been shown to lower the risk of developing IBD [31]. Thus, the potential benefits of favorable metabolic factors could be obscured by both behavioral changes and the effects of medication use, especially in this relatively older population with high prevalent metabolic disorders. Nevertheless, further studies are needed to validate our hypothesis. A recent cohort study using UK Biobank data revealed that an unfavorable lifestyle (including 5 factors: BMI, smoking, diets, PA, and sleep duration) was significantly associated with increased risks of UC (HR: 1.98; 95% CI: 1.73–2.27) and CD (HR: 1.94; 95% CI: 1.61–2.33) [32]. However, these studies were limited to single or partial combinations of LE8 metrics. The influence of other LE8 factors disruptions, like blood pressure and serum HDL-C [33, 34], on IBD risk has also been established. Moreover, many studies adopted a binary scoring system (0 or 1 point) for health behaviors [32], which might oversimplify complex lifestyle patterns. In contrast, the LE8 scores applies a more nuanced and continuous scale (0–100 points for each component), offering greater sensitivity in detecting interindividual differences and intraindividual change. To the best of our knowledge, the present study for the first time applied LE8, the new CVH score, to the risk of IBD. Our results showed that both moderate and high levels of CVH were associated with lower risk of IBD. These findings provide robust support for the promotion of LE8-based CVH in the prevention of IBD. Notably, every 10-point increase was significantly associated with lower risks of IBD (HR: 0.90; 95% CI: 0.86–0.94), UC (HR: 0.90; 95% CI: 0.85–0.95), and CD (HR: 0.88; 95% CI: 0.82–0.95). These findings encourage individuals to progressively improve their health behaviors and lifestyle factors, highlighting the value of gradual changes over attempting too many alterations simultaneously [35]. In other words, even small, incremental improvements are beneficial and preferable to no change at all.

Although the precise mechanism between LE8 and IBD remains to be fully elucidated, the individual components of LE8 have been extensively studied. First, substantial evidence has demonstrated that the unhealthy categories in LE8 metrics, such as the lack of PA, overweight and obesity, smoking, poor diets, sleep disturbances, high BP, and dysregulation of serum lipids and blood glucose, can disrupt immune homeostasis and contribute to the induction and exacerbation of inflammatory processes [13, 36–38]. Chronic inflammation is a well-established risk factor in the development of IBD [1]. Second, gut microbiota, which plays a key role in the pathogenesis of IBD [39], can be positively influenced by appropriate PA, smoking cessation, and particularly, by maintaining healthy diets [40–43]. Modifications in these health behaviors can modify the composition, diversity, and metabolic capacity of the gut microbiota, and may thus enhance gut and systemic immune function and thereby prevent the onset of IBD [40–43]. In addition, adherence to these healthy behaviors in LE8 is associated with decreased risk of mood disorders [44–47], which are also identified as risk factors for IBD [13]. Taken together, this evidence lend support to the observed association between the LE8 scores and incident IBD. Future randomized clinical trials are needed to validate our findings.

To our knowledge, this is the first study to explore the association between LE8-defined CVH, polygenic risk, and the incidence of IBD. Our findings are partially in line with previous research, which reported no significant interaction between lifestyle and genetic susceptibility to UC or CD [48]. No significant interactions between genetic risk, LE8 scores, and the incidence of UC or CD were observed in the present study. Our results highlight the potential of enhancing CVH by following the LE8 guidelines as a universally beneficial approach for the prevention of IBD, applicable to individuals irrespective of their genetic predisposition to this condition.

The major strengths of this study include the population-based cohort study design, large sample size, a careful consideration of potential confounding factors, and a series of sensitivity analyses. However, it is important to acknowledge certain limitations. First, despite adjusting for multiple confounders, the potential residual confounders cannot be entirely ruled out. Second, information on dietary assessment, PA, nicotine exposure, and sleep duration within LE8 was self-reported, which may lead to information bias and misclassification. Third, data on changes of LE8 metrics over time were not available, so we were unable to assess the association between dynamic changes of LE8 and IBD incidence. Future research with longitudinal LE8 measurements is needed to evaluate the impact of these changes on IBD risk. Fourth, the UK Biobank is not representative of the population in other respects with evidence of a ‘selection’ or ‘healthy volunteer’ bias [49, 50]. Fifth, due to the nature of observational study, causal inference cannot be made in this study. To mitigate this limitation, we excluded participants who developed IBD within the first two years of follow-up, and this did not change the observed associations. Sixth, the identification of incident IBD cases was ascertained through hospital inpatient records and death registry, which may result in missed cases. However, misclassification errors were likely to have biased these findings towards the null and would underestimate the true association between LE8-defined CVH and IBD risk. Lastly, our analyses were limited to individuals of European ancestry and an older population, thus the generalizability of our findings to other ethnicities and younger age groups may be limited.

### Conclusions

In summary, our results reveals that better LE8-defined CVH was associated with lower risk of IBD, UC, and CD, irrespective of genetic predisposition. These findings underscore the importance of adherence to LE8 guidelines for maintaining CVH as a potential strategy in the prevention of IBD.

### Electronic supplementary material

Below is the link to the electronic supplementary material.

Supplementary Material 1

Supplementary Material 2

## Data Availability

Data are available in a public, open access repository. This research has been conducted using the UK Biobank Resource under Application Number 63454. The UK Biobank data are available on application to the UK Biobank (www.ukbiobank.ac.uk/).

## Abbreviations

AHA: American Heart Association
BMI: body mass index
BP: blood pressure
CD: Crohn’s disease
CI: confidence interval
CVD: cardiovascular disease
CVH: cardiovascular health
FFQ: food frequency questionnaire
HDL: high density lipoprotein
HDS: healthy diet score
HR: hazard ratio
IBD: Inflammatory bowel diseases
ICD: International Classification of Diseases
LE8: Life’s Essential 8
PA: physical activity
PRS: polygenic risk scores
SD: standard deviation
TDI: Townsend Deprivation Index
UC: ulcerative colitis
