# Supplementary material for: Life’s essential 8, genetic susceptibility, and risk of inflammatory bowel diseases: a population-based cohort study
Source: Int J Behav Nutr Phys Act. 2024 Jul 2;21:66. doi: 10.1186/s12966-024-01617-3 (PMC11221134; doi:10.1186/s12966-024-01617-3)
Supplement: Supplementary file 2 — Supplementary Material 2 [file 12966_2024_1617_MOESM2_ESM.docx]

**Supplementary appendix**

**Supplementary Figure 1**. Flow chart for the selection of the study sample

**Supplementary Table 1**. Healthy diet score using touchscreen questionnaire in the UK biobank study

**Supplementary Table 2**. Quantitative assessment of Life’s Essential 8 (LE8)

**Supplementary Table 3**. Associations between the eight components of LE8 and the risks of IBD, UC, and CD

**Supplementary Table 4**. Associations of PRS with the risks of UC and CD

**Supplementary Table 5**. Hazard ratios (95% confidence intervals) for IBD, UC, and CD according to Life’s Essential 8 stratified by sex and age

**Supplemental Table 6**. Associations of Life’s Essential 8 with the risk of IBD, UC, and CD after the exclusion of participants who were diagnosed with cancer at baseline

**Supplemental Table 7**. Associations of Life’s Essential 8 with the risk of IBD, UC, and CD after the exclusion of participants who developed IBD during the first 2 years of follow-up

**Supplemental Table 8**. Associations of Life’s Essential 8 scores with the risk of IBD, UC, and CD using competing risk regression

**
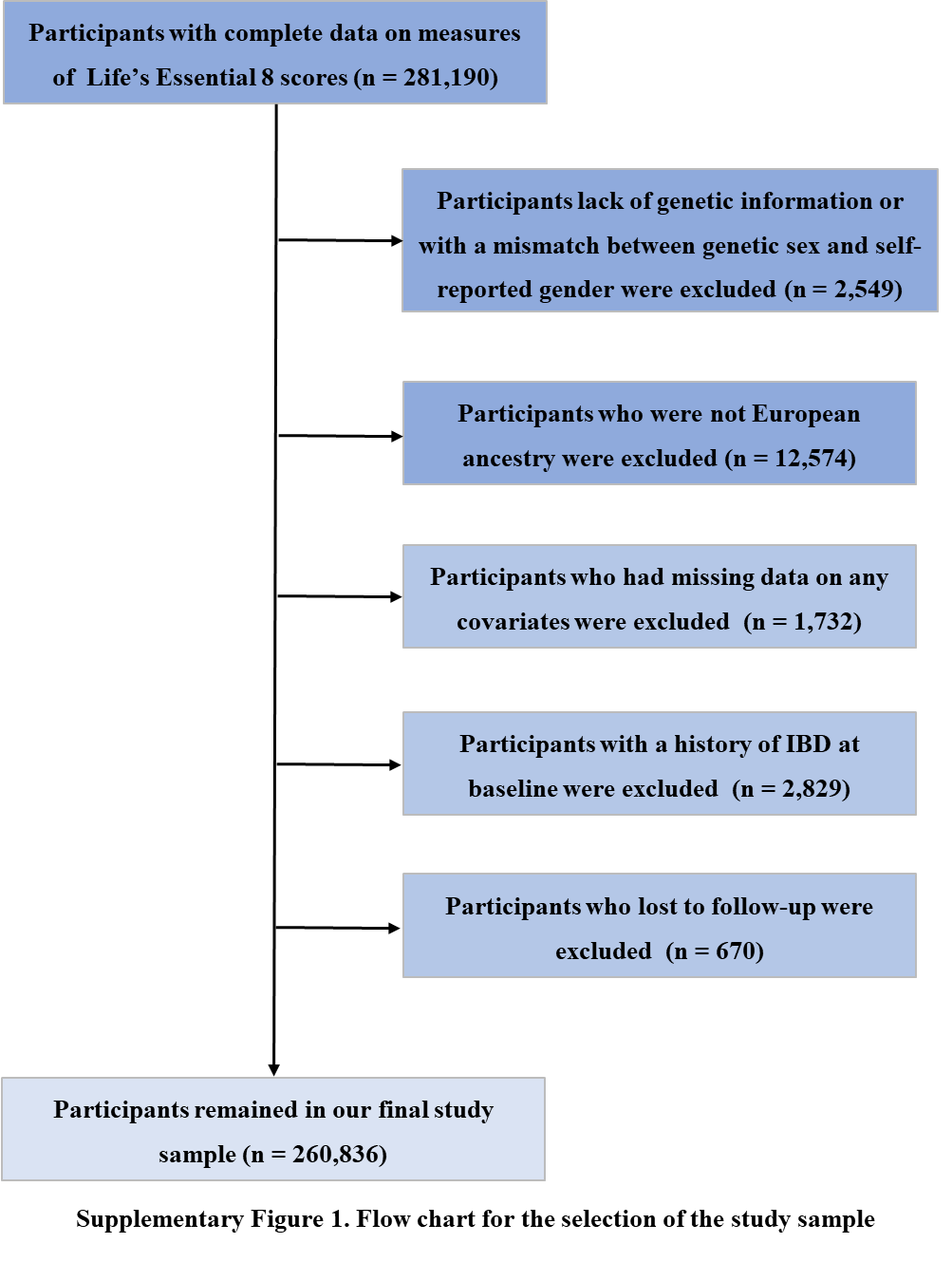
**

**Supplementary Table 1.** Healthy diet score using touchscreen questionnaire in the UK biobank study.

| **Food group/nutrient item** | **UKB fields used** | **Definition of meeting criterion** | **Amount per serving** |
| --- | --- | --- | --- |
| **Consume more Fruits, fresh or dried** | 1309 and 1319 | ≥3 servings/d  Including fresh and dried fruits | 1309 – 1 piece  1319 – 3 pieces |
| **Vegetables, salad/cooked** | 1289 and 1299 | ≥3 servings/d  Including salad, raw and cooked | 1289 – 8 tablespoons  1299 – 12 tablespoons |
| **Whole grains** | 1438 and 1448  1458 and 1468 | ≥3 servings/d  Daily slices of whole meal or wholegrain bread (servings/d), convert from weekly slices.  Daily bowls of whole wheat cereal as servings/d (bran cereal, biscuit cereal, oat cereal and muesli), convert from weekly bowls. | 1438/1448 – 1 slice/d  1458/1468 – 1 bowl/d |
| **Fish shellfish** | 1329 and 1339 | ≥2 servings/wk | Once/wk |
| **Dairy products** | 6144, 1408 and 1418 | Sum weekly frequencies to obtain total servings/wk.  Reporting consumption of one milk item or eating cheese once a  day to meet the 2-3 servings/d criterion. | 1408 – once/d  1418 – consumption of any type of milk |
| **Vegetable oil** | 2654 | Reporting use of olive oil or polyunsaturated/sunflower oil (yes=1, 0=no) |  |
| **Consume less Refined grains, starches, added sugars** | 1438 and 1448  1458 and 1468 | Reporting use of white, brown, other bread or other cereals; <1.5 servings/d | 1438/1448 – 1 slice/d  1458/1468 – 1 bowl/d |
| **Processed meats** | 1349 and 3680 | Once/wk or less would meet the criterion. |  |
| **Unprocessed red meats** | 1369, 1379, 1389, and 3680 | Summation of frequency of consumption across three types of red meats (lamb/mutton, beef or pork). <3 on the summation corresponds to the criterion of <1-2 9 servings per week. |  |
| **Industrial trans fat** | 1428 and 2654 | Reporting use of Flora Pro-Active/Benecol spread, soft margarine -, olive oil based -, polyunsaturated/ sunflower oil based -, other low/reduced fat spread or never use spread would meet the criterion |  |
| **Sugar-sweetened beverages** | 6144 | Never eat sugar or food/drink containing sugar would meet the criterion |  |
| **Sodium** | 1478 | Salt added to food, never or rarely would meet the criterion |  |

**Supplementary Table 2.** Quantitative assessment of Life’s Essential 8 (LE8).

| LE8 metric | Method of measurement | Quantification of LE8 metric | Score and classification |
| --- | --- | --- | --- |
| Diet | Measurement: Healthy Diet Score | Scoring:  Points diets score (points)  100 8–10  80 6–7  50 4–5  25 2–3  0 0–1 | The LE8 score is scaled from 0 to 100 points, calculated as the unweighted average of all 8 component metric scores. In the present study, overall LE8 scores of 80 to 100 are considered High CVH; 50 to 79, Moderate CVH; and 0 to 49 points, low CVH. |
| Physical activity (PA) | Self-reported minutes of moderate or vigorous PA per week (1 minute of vigorous physical activity is equivalent to 2 minutes of moderate physical activity). | Scoring:  Points Minutes  100 ≥150  90 120–149  80 90–119  60 60–89  40 30–59  20 1–29  0 0 |  |
| Tobacco/nicotine exposure | Self-reported use of cigarettes; or secondhand smoke exposure (Participants were asked “Does anyone in your household smoke”. Secondhand smoke exposure was defined as if participants’ responses are “yes, one household member smokes” or “Yes, more than one household member smokes”). | Scoring:  Points Status  100 Never smoker  75 Former smoker, quit ≥5 y  50 Former smoker, quit 1–<5 y  25 Former smoker, quit <1 y  0 Current smoker  Subtract 20 points (unless score is 0) for living with active indoor smoker in home. Furthermore, only individuals who indicated they “smoked on most or all days in the past” have access to information on the precise time to quit smoking. We consider participants who indicated “smoked occasionally in the past” as equivalent to “Former smoker, quit 1–<5 years”; We consider the participants who indicated “just tried once or twice in the past” as equivalent to “Former smoker, quit ≥ 5 years”. |  |
| Sleep health | Self-reported average hours of sleep per night | Scoring:  Points Level  100 7–<9  90 9–<10  70 6–<7  40 5–<6 or ≥10  20 4–<5  0 <4 |  |
| Body mass index | Measurement: Body weight (kilograms)  divided by height squared (meters  squared) | Scoring:  Points Level  100 <25  70 25.0–29.9  30 30.0–34.9  15 35.0–39.9  0 ≥40.0 |  |
| Blood lipids (non-HDL cholesterol) | Measurement: Plasma total and HDL cholesterol with calculation of non–HDL cholesterol | Metric: Non–HDL cholesterol (mg/dL)  Scoring:  Points Level  100 <130  60 130–159  40 160–189  20 190–219  0 ≥220  If drug-treated level, subtract 20 points |  |
| Blood glucose | Measurement: HbA1c (%) and history of diabetes | Metric: HbA1c (%)  Scoring:  Points Level  100 No history of diabetes and HbA1c < 5.7  60 No diabetes and HbA1c 5.7–6.4 (prediabetes)  40 Diabetes with HbA1c <7.0  30 Diabetes with HbA1c 7.0–7.9  20 Diabetes with HbA1c 8.0–8.9  10 Diabetes with Hb A1c 9.0–9.9  0 Diabetes with HbA1c ≥10.0 |  |
| Blood pressure (BP) | Measurement: Appropriately measured systolic and diastolic blood pressures (Average values of systolic and diastolic BP blood pressure were utilized, and automated readings were preferred. In case where automated readings were unavailable, manual readings were employed.) | Metric: Systolic and diastolic BPs (mm Hg)  Scoring:  Points Level  100 <120/<80 (optimal)  75 120–129/<80 (elevated)  50 130–139 or 80–89 (stage 1 hypertension)  25 140–159 or 90–99  0 ≥160 or ≥100  Subtract 20 points if treated level |  |

| **Supplemental Table 3.** Associations between the eight components of LE8 and the risks of IBD, UC, and CD ^a^ | | | | |
| --- | --- | --- | --- | --- |
| LE8 components scores | SD value | Each SD increase | | |
|  |  | IBD | UC | CD |
| Dietary quality | 23.8 | 0.94 (0.89, 0.99) ^b^ | 0.95 (0.90, 1.01) | 0.92 (0.84, 1.00) |
| Physical activity | 36.4 | 0.93 (0.89, 0.98) | 0.96 (0.90, 1.01) | 0.88 (0.81, 0.95) |
| Sleep health | 18.2 | 0.92 (0.88, 0.96) | 0.94 (0.89, 0.99) | 0.87 (0.81, 0.94) |
| Nicotine exposure | 29.1 | 0.88 (0.84, 0.92) | 0.89 (0.85, 0.95) | 0.83 (0.77, 0.90) |
| Body mass index | 28.3 | 0.93 (0.89, 0.98) | 0.92 (0.87, 0.98) | 0.95 (0.87, 1.04) |
| Blood lipids | 29.0 | 1.16 (1.10, 1.22) | 1.11 (1.04, 1.17) | 1.26 (1.16, 1.38) |
| Blood glucose | 13.0 | 0.95 (0.91, 0.99) | 0.95 (0.90, 0.99) | 0.99 (0.91, 1.07) |
| Blood pressure | 32.3 | 1.02 (0.96, 1.08) | 0.99 (0.93, 1.06) | 1.06 (0.97, 1.17) |
| ^a^ Abbreviations: CD, Crohn's disease; IBD, inflammatory bowel disease; LE8, Life’s Essential 8; PRS, polygenic risk scores; SD, standard deviation; UC, ulcerative colitis. | | | | |
| ^b^ Hazard ratio (95% confidence interval) (all such values). Multivariable Cox proportional hazards regression was adjusted for age (continuous), sex (male or female), Townsend Deprivation Index (categorical, quartiles), drinking status (current, previous, or never), education levels (low, medium, or high), depression (yes or no), UC-PRS (<median or ≥median, only in UC), CD-PRS (<median or ≥median, only in CD), genotyping batch (only in UC and CD), the first 10 principal components of genetics (only in UC and CD), and each SD increase in the overall LE8 scores calculated by the other remaining 7 components. | | | | |
|  |  |  |  |  |
|  |  |  |  |  |
|  |  |  |  |  |

| **Supplementary Table 4**. Associations of PRS with the risks of UC and CD ^a^ | | | |  |
| --- | --- | --- | --- | --- |
|  | PRS | | |  |
|  | < Median | ≥Median | Each SD increase |  |
| UC |  |  |  |  |
| Cases, n | 346 | 724 | 1,070 |  |
| Person-years | 1,537,115 | 1,534,683 | 3,071,798 |  |
| Model 1 ^c^ | 1.00 (reference) | 2.09 (1.84, 2.38) ^b^ | 1.63 (1.54, 1.73) |  |
| Model 2 ^d^ | 1.00 (reference) | 2.09 (1.84, 2.38) | 1.63 (1.54, 1.73) |  |
| Model 3 ^e^ | 1.00 (reference) | 2.07 (1.82, 2.36) | 1.63 (1.53, 1.73) |  |
|  |  |  |  |  |
| CD |  |  |  |  |
| Cases, n | 170 | 332 | 502 |  |
| Person-years | 1,545,898 | 1,528,985 | 3,074,883 |  |
| Model 1 | 1.00 (Ref) | 1.97 (1.64, 2.37) | 1.65 (1.50, 1.82) |  |
| Model 2 | 1.00 (Ref) | 1.97 (1.64, 2.37) | 1.66 (1.50, 1.82) |  |
| Model 3 | 1.00 (Ref) | 1.97 (1.64, 2.37) | 1.67 (1.51, 1.84) |  |
| ^a^ Abbreviations: CD, Crohn's disease; LE8, Life’s Essential 8; PRS, polygenic risk scores; SD, standard deviation; UC, ulcerative colitis. | | | |  |
|  |  |  |  |  |
| ^b^ Hazard ratio (95% confidence interval) (all such values). | | | |  |
| ^c^ Model 1 was a crude model. | | | |  |
| ^d^ Model 2 was adjusted for age (continuous) and sex (male or female). | | | |  |
| ^e^ Model 3 was further adjusted for Townsend Deprivation Index (categorical, quartiles), drinking status (current, previous, or never), education levels (low, medium, or high), depression (yes or no), genotyping batch, the first 10 principal components of genetics, LE8 scores (low [0-49], moderate [50-79], or high [80-100]), UC-PRS (continuous, only in UC), and CD-PRS (continuous, only in CD). | | | |  |
|  |  |  |  |  |

| **Supplementary Table 5**. Hazard ratios (95% confidence intervals) for IBD, UC, and CD according to Life’s Essential 8 scores stratified by sex and age ^a^ | | | | | |  |
| --- | --- | --- | --- | --- | --- | --- |
|  |  |  |  |  |  |  |
|  | Life’s Essential 8 | | | Per 10-point increase | *P* for interaction ^b^ |  |
|  | Low (0-49) | Moderate (50-79) | High (80-100) |  |  |  |
| IBD |  |  |  |  |  |  |
| Sex |  |  |  |  | 0.06 |  |
| Males | 1.00 (reference) | 1.01 (0.77, 1.32) ^c^ | 0.81 (0.58, 1.15) | 0.93 (0.87, 0.98) |  |  |
| Females | 1.00 (reference) | 0.61 (0.46, 0.81) | 0.50 (0.36, 0.70) | 0.87 (0.81, 0.93) |  |  |
| Age (years) |  |  |  |  | 0.54 |  |
| <55 | 1.00 (reference) | 0.75 (0.55, 1.03) | 0.60 (0.41, 0.86) | 0.88 (0.82, 0.94) |  |  |
| ≥55 | 1.00 (reference) | 0.85 (0.66, 1.09) | 0.69 (0.51, 0.94) | 0.91 (0.86, 0.96) |  |  |
|  |  |  |  |  |  |  |
| UC |  |  |  |  |  |  |
| Sex |  |  |  |  | 0.21 |  |
| Males | 1.00 (reference) | 1.02 (0.75, 1.40) | 0.80 (0.53, 1.19) | 0.91 (0.85, 0.98) |  |  |
| Females | 1.00 (reference) | 0.78 (0.54, 1.13) | 0.59 (0.38, 0.90) | 0.89 (0.82, 0.97) |  |  |
| Age (years) |  |  |  |  | 0.56 |  |
| <55 | 1.00 (reference) | 0.77 (0.53, 1.11) | 0.61 (0.40, 0.94) | 0.88 (0.81, 0.96) |  |  |
| ≥55 | 1.00 (reference) | 1.03 (0.75, 1.41) | 0.75 (0.51, 1.10) | 0.91 (0.85, 0.97) |  |  |
|  |  |  |  |  |  |  |
| CD |  |  |  |  |  |  |
| Sex |  |  |  |  | 0.15 |  |
| Males | 1.00 (reference) | 0.98 (0.60, 1.59) | 0.78 (0.42, 1.46) | 0.97 (0.86, 1.09) |  |  |
| Females | 1.00 (reference) | 0.42 (0.28, 0.62) | 0.40 (0.25, 0.64) | 0.82 (0.74, 0.91) |  |  |
| Age (years) |  |  |  |  | 0.28 |  |
| <55 | 1.00 (reference) | 0.59 (0.36, 0.96) | 0.43 (0.24, 0.77) | 0.82 (0.73, 0.92) |  |  |
| ≥55 | 1.00 (reference) | 0.64 (0.43, 0.95) | 0.65 (0.40, 1.06) | 0.92 (0.83, 1.02) |  |  |
| ^a^ Abbreviations: CD, Crohn's disease; IBD, inflammatory bowel disease; PRS, polygenic risk scores; UC, ulcerative colitis. | | | | | |  |
| ^b^ *P* for interaction was assessed by adding the multiplicative interaction terms of Life’s Essential 8 scores with stratifying variables in the models. | | | | | |  |
| ^c^ Hazard ratio (95% confidence interval) (all such values). Multivariable Cox proportional hazards regression was adjusted for age (continuous), sex (male or female), Townsend Deprivation Index (categorical, quartiles), drinking status (current, previous, or never), education levels (low, medium, or high), depression (yes or no), UC-PRS (<median or ≥median, only in UC), CD-PRS (<median or ≥median, only in CD), genotyping batch (only in UC and CD), and the first 10 principal components of genetics (only in UC and CD). | | | | | |  |
|  |  |  |  |  |  |  |

| **Supplemental Table 6**. Associations of Life’s Essential 8 scores with the risks of IBD, UC, and CD after the exclusion of participants who were diagnosed with cancer at baseline ^a^ | | | | |  |
| --- | --- | --- | --- | --- | --- |
|  |  |  |  |  |  |
|  | Life’s Essential 8 | | | Per 10-point increase |  |
|  | Low (0-49) | Moderate (50-79) | High (80-100) |  |  |
| **IBD** |  |  |  |  |  |
| Cases/participants, n | 101/13,450 | 1,057/182,236 | 176/42,408 | 1,334/238,094 |  |
| Person-years | 156,177 | 2,149,271 | 501,927 | 2,807,375 |  |
| Model 1 ^c^ | 1.00 (reference) | 0.76 (0.62, 0.93) ^b^ | 0.54 (0.42, 0.69) | 0.86 (0.82, 0.90) |  |
| Model 2 ^d^ | 1.00 (reference) | 0.77 (0.63, 0.94) | 0.59 (0.46, 0.76) | 0.88 (0.84, 0.92) |  |
| Model 3 ^e^ | 1.00 (reference) | 0.81 (0.66, 1.00) | 0.64 (0.50, 0.82) | 0.89 (0.85, 0.94) |  |
|  |  |  |  |  |  |
| **UC** |  |  |  |  |  |
| Cases/participants, n | 67/13,450 | 775/182,236 | 115/42,408 | 957/238,094 |  |
| Person-years | 156,393 | 2,150,829 | 502,337 | 2,809,559 |  |
| Model 1 | 1.00 (reference) | 0.84 (0.66, 1.08) | 0.53 (0.40, 0.72) | 0.85 (0.81, 0.90) |  |
| Model 2 | 1.00 (reference) | 0.86 (0.67, 1.10) | 0.60 (0.44, 0.82) | 0.87 (0.83, 0.92) |  |
| Model 3 | 1.00 (reference) | 0.92 (0.71, 1.18) | 0.66 (0.48, 0.89) | 0.89 (0.84, 0.94) |  |
|  |  |  |  |  |  |
| **CD** |  |  |  |  |  |
| Cases/participants, n | 42/13,450 | 329/182,236 | 66/42,408 | 437/238,094 |  |
| Person-years | 156,464 | 2,153,393 | 502,547 | 2,812,404 |  |
| Model 1 | 1.00 (reference) | 0.57 (0.41, 0.78) | 0.49 (0.33, 0.72) | 0.86 (0.79, 0.93) |  |
| Model 2 | 1.00 (reference) | 0.57 (0.41, 0.78) | 0.49 (0.33, 0.73) | 0.86 (0.79, 0.93) |  |
| Model 3 | 1.00 (reference) | 0.62 (0.44, 0.85) | 0.55 (0.37, 0.82) | 0.88 (0.81, 0.96) |  |
| ^a^ Abbreviations: CD, Crohn's disease; IBD, inflammatory bowel disease; PRS, polygenic risk scores; UC, ulcerative colitis. | | | | |  |
| ^b^ Hazard ratio (95% confidence interval) (all such values). | | | | |  |
| ^c^ Model 1 was a crude model. | | | | |  |
| ^d^ Model 2 was adjusted for age (continuous) and sex (male or female). | | | | |  |
| ^e^ Model 3 was further adjusted for Townsend Deprivation Index (categorical, quartiles), drinking status (current, previous, or never), education levels (low, medium, or high), depression (yes or no), UC-PRS (<median or ≥median, only in UC), CD-PRS (<median or ≥median, only in CD), genotyping batch (only in UC and CD), and the first 10 principal components of genetics (only in UC and CD). | | | | |  |
|  |  |  |  |  |  |
|  |  |  |  |  |  |

| **Supplemental Table 7**. Associations of Life’s Essential 8 scores with the risks of IBD, UC, and CD after the exclusion of participants who developed IBD during the first 2 years of follow-up ^a^ | | | | |  |
| --- | --- | --- | --- | --- | --- |
|  |  |  |  |  |  |
|  | Life’s Essential 8 | | | Per 10-point increase |  |
|  | Low (0-49) | Moderate (50-79) | High (80-100) |  |  |
| **IBD** |  |  |  |  |  |
| Cases/participants, n | 98/14,883 | 1,046/199,764 | 166/45,999 | 1,310/260,646 |  |
| Person-years | 172,108 | 2,352,529 | 544,508 | 3,069,145 |  |
| Model 1 ^c^ | 1.00 (reference) | 0.78 (0.63, 0.96) ^b^ | 0.53 (0.42, 0.68) | 0.86 (0.82, 0.90) |  |
| Model 2 ^d^ | 1.00 (reference) | 0.79 (0.64, 0.97) | 0.58 (0.45, 0.75) | 0.87 (0.83, 0.92) |  |
| Model 3 ^e^ | 1.00 (reference) | 0.84 (0.68, 1.03) | 0.63 (0.49, 0.82) | 0.89 (0.85, 0.93) |  |
|  |  |  |  |  |  |
| **UC** |  |  |  |  |  |
| Cases/participants, n | 68/14,883 | 754/199,764 | 114/45,999 | 936/260,646 |  |
| Person-years | 172,261 | 2,353,938 | 544,784 | 3,070,983 |  |
| Model 1 | 1.00 (reference) | 0.81 (0.63, 1.04) | 0.53 (0.39, 0.71) | 0.85 (0.80, 0.90) |  |
| Model 2 | 1.00 (reference) | 0.82 (0.64, 1.06) | 0.60 (0.44, 0.81) | 0.87 (0.82, 0.92) |  |
| Model 3 | 1.00 (reference) | 0.89 (0.69, 1.14) | 0.66 (0.49, 0.90) | 0.89 (0.84, 0.94) |  |
|  |  |  |  |  |  |
| **CD** |  |  |  |  |  |
| Cases/participants, n | 39/14,883 | 334/199,764 | 58/45,999 | 431/260,646 |  |
| Person-years | 172,370 | 2,356,008 | 545,031 | 3,073,409 |  |
| Model 1 | 1.00 (reference) | 0.62 (0.45, 0.87) | 0.47 (0.31, 0.70) | 0.85 (0.78, 0.92) |  |
| Model 2 | 1.00 (reference) | 0.62 (0.45, 0.87) | 0.46 (0.31, 0.70) | 0.84 (0.78, 0.91) |  |
| Model 3 | 1.00 (reference) | 0.68 (0.49, 0.95) | 0.52 (0.35, 0.80) | 0.87 (0.80, 0.94) |  |
| ^a^ Abbreviations: CD, Crohn's disease; IBD, inflammatory bowel disease; PRS, polygenic risk scores; UC, ulcerative colitis. | | | | |  |
| ^b^ Hazard ratio (95% confidence interval) (all such values). | | | | |  |
| ^c^ Model 1 was a crude model. | | | | |  |
| ^d^ Model 2 was adjusted for age (continuous) and sex (male or female). | | | | |  |
| ^e^ Model 3 was further adjusted for Townsend Deprivation Index (categorical, quartiles), drinking status (current, previous, or never), education levels (low, medium, or high), depression (yes or no), UC-PRS (<median or ≥median, only in UC), CD-PRS (<median or ≥median, only in CD), genotyping batch (only in UC and CD), and the first 10 principal components of genetics (only in UC and CD). | | | | |  |
|  |  |  |  |  |  |
|  |  |  |  |  |  |

| **Supplemental Table 8**. Associations of Life’s Essential 8 scores with the risks of IBD, UC, and CD using competing risk regression ^a^ | | | | |  |
| --- | --- | --- | --- | --- | --- |
|  | Life’s Essential 8 | | | Per 10-point increase |  |
|  | Low (0-49) | Moderate (50-79) | High (80-100) |  |  |
| **IBD** |  |  |  |  |  |
| Cases/participants, n | 114/14,899 | 1,187/199,905 | 199/46,032 | 1,500/260,836 |  |
| Person-years | 172,127 | 2,352,673 | 544,541 | 3,069,341 |  |
| Model 1 ^c^ | 1.00 (reference) | 0.79 (0.65, 0.96) ^b^ | 0.58 (0.46, 0.73) | 0.87 (0.84, 0.91) |  |
| Model 2 ^d^ | 1.00 (reference) | 0.80 (0.60, 0.97) | 0.63 (0.50, 0.80) | 0.89 (0.85, 0.93) |  |
| Model 3 ^e^ | 1.00 (reference) | 0.85 (0.70, 1.03) | 0.68 (0.54, 0.86) | 0.91 (0.87, 0.95) |  |
|  |  |  |  |  |  |
| **UC** |  |  |  |  |  |
| Cases/participants, n | 75/14,899 | 862/199,905 | 133/46,032 | 1,070/260,836 |  |
| Person-years | 172,357 | 2,354,472 | 544,969 | 3,071,798 |  |
| Model 1 | 1.00 (reference) | 0.87 (0.69, 1.10) | 0.59 (0.45, 0.78) | 0.87 (0.83, 0.91) |  |
| Model 2 | 1.00 (reference) | 0.89 (0.70, 1.13) | 0.66 (0.50, 0.89) | 0.89 (0.85, 0.94) |  |
| Model 3 | 1.00 (reference) | 0.96 (0.75, 1.21) | 0.73 (0.54, 0.97) | 0.91 (0.86, 0.96) |  |
|  |  |  |  |  |  |
| **CD** |  |  |  |  |  |
| Cases/participants, n | 48/14,899 | 380/199,905 | 74/46,032 | 502/260,836 |  |
| Person-years | 172,449 | 2,357,183 | 545,251 | 3,074,883 |  |
| Model 1 | 1.00 (reference) | 0.60 (0.44, 0.81) | 0.51 (0.36, 0.74) | 0.87 (0.80, 0.94) |  |
| Model 2 | 1.00 (reference) | 0.60 (0.44, 0.81) | 0.51 (0.35, 0.75) | 0.87 (0.80, 0.94) |  |
| Model 3 | 1.00 (reference) | 0.65 (0.48, 0.88) | 0.57 (0.40, 0.83) | 0.89 (0.82, 0.96) |  |
| ^a^ Abbreviations: CD, Crohn's disease; IBD, inflammatory bowel disease; PRS, polygenic risk scores; UC, ulcerative colitis. | | | | |  |
| ^b^ Hazard ratio (95% confidence interval) (all such values). | | |  |  |  |
| ^c^ Model 1 was a crude model. | |  |  |  |  |
| ^d^ Model 2 was adjusted for age (continuous) and sex (male or female). | | | |  |  |
| ^e^ Model 3 was further adjusted for Townsend Deprivation Index (categorical, quartiles), drinking status (current, previous, or never), education levels (low, medium, or high), depression (yes or no), UC-PRS (<median or ≥median, only in UC), CD-PRS (<median or ≥median, only in CD), genotyping batch (only in UC and CD), and the first 10 principal components of genetics (only in UC and CD). | | | | |  |
|  |  |  |  |  |  |
